# A Model-Based Analysis of Culture-Dependent Phenotypes of mESCs

**DOI:** 10.1371/journal.pone.0092496

**Published:** 2014-03-18

**Authors:** Maria Herberg, Tüzer Kalkan, Ingmar Glauche, Austin Smith, Ingo Roeder

**Affiliations:** 1 Institute for Medical Informatics and Biometry, Medical Faculty Carl Gustav Carus, Technische Universität Dresden, Dresden, Germany; 2 Wellcome Trust-Medical Research Council Cambridge Stem Cell Institute, University of Cambridge, Cambridge, United Kingdom; German Cancer Research Center, Germany

## Abstract

Mouse embryonic stem cells (mESCs) can be maintained in a proliferative and undifferentiated state over many passages (self-renewal) while retaining the potential to give rise to every cell type of the organism (pluripotency). Autocrine FGF4/Erk signalling has been identified as a major stimulus for fate decisions and lineage commitment in these cells. Recent findings on serum-free culture conditions with specific inhibitors (known as 2i) demonstrate that the inhibition of this pathway reduces transcription factor heterogeneity and is vital to maintain ground state pluripotency of mESCs.

We suggest a novel mathematical model to explicitly integrate FGF4/Erk signalling into an interaction network of key pluripotency factors (namely Oct4, Sox2, Nanog and Rex1). The envisaged model allows to explore whether and how proposed mechanisms and feedback regulations can account for different expression patterns in mESC cultures. We demonstrate that an FGF4/Erk-mediated negative feedback is sufficient to induce molecular heterogeneity with respect to Nanog and Rex1 expression and thus critically regulates the propensity for differentiation and the loss of pluripotency. Furthermore, we compare simulation results on the transcription factor dynamics in different self-renewing states and during differentiation with experimental data on a Rex1GFPd2 reporter cell line using flow cytometry and qRT-PCR measurements. Concluding from our results we argue that interaction between FGF4/Erk signalling and Nanog expression qualifies as a key mechanism to manipulate mESC pluripotency. In particular, we infer that ground state pluripotency under 2i is achieved by shifting stable expression pattern of Nanog from a bistable into a monostable regulation impeding stochastic state transitions. Furthermore, we derive testable predictions on altering the degree of Nanog heterogeneity and on the frequency of state transitions in LIF/serum conditions to challenge our model assumptions.

## Introduction

Mouse embryonic stem cells (mESCs) are pluripotent cell lines derived from the inner cell mass (ICM) of a blastocyst stage mouse embryo [1], [2]. Under appropriate culture conditions mESCs can be maintained in an undifferentiated state over many passages while keeping the capacity to contribute to embryonic development *in vivo*, a property termed self-renewal. The best-known factor to promote self-renewal is the cytokine LIF (leukaemia inhibitory factor) [3]. LIF and mESCs themselves activate the differentiation-inducing MAPK/Erk (mitogen-activated protein kinases) signalling pathway [4], [5]. To compensate this effect, ID (inhibitor of differentiation) proteins have to be activated through serum factors or BMPs (bone morphogenetic proteins). Overall, the maintenance of mESC pluripotency is considered to rely on a multi-layered activation and repression of transcriptional determinants by extrinsic regulators. Although many factors involved in this interplay have been identified, critical interactions and underlying dynamic processes driving fate decisions have yet to be fully defined.

The transcription factors (TFs) Oct4, Sox2 and Nanog are key elements of an intrinsic, self-organizing network capable to maintain the pluripotent state of mESCs [6], [7], [8]. Co- and autoregulatory links among these genes have been proposed to stabilize expression levels of pluripotency factors, whereas lineage-specific genes are confined by mutual antagonisms [7], [9], [10]. The induction of cell differentiation is often considered as a switch-like transition [11], [12], [13]. However, dose-dependent phenotypes and variable expression levels require a revision of this concept related to mESC development. In particular, it has been demonstrated that defined levels of the TF Oct4 govern distinct cell fates [14], [15], [16]. While continuous expression of Oct4 at a certain level is required to sustain self-renewal, an increase of Oct4 causes differentiation into primitive endoderm and mesoderm. In contrast, acute repression of Oct4 leads to differentiation into the trophectoderm linage [15], [17]. Contrasting with the apparent homogeneity of Oct4 levels in pluripotent mESCs, expression levels of the pluripotency factor Nanog have been identified as heterogeneous and variable [18], [19]. In fact, Oct4-positive mESCs can be subdivided into Nanog-high (NH) and Nanog-low (NL) cells. After sorting these two cell populations, the original bimodal distribution of NH and NL cells is gradually reestablished [18], [20]. Importantly this phenomenon can be recapitulated on a single cell level [18]. Although all Oct4-positive mESCs can retain pluripotency, NL cells are significantly more prone to differentiation than NH cells [18], [21]. A similar pattern has been observed for the TF Rex1 (gene name *Zfp42*) [22]. Rex1 is a reliable marker for undifferentiated mESCs and described as downstream target of Nanog, Oct4 and Sox2 [22], [23], [24]. Subdividing mESCs into Rex1-high (RH) and Rex1-low (RL) cells, both fractions show different gene expression patterns and differentiation capacities [22], [25].

It is of particular interest that the proportion of high and low expressing cells depends on the composition of the culture conditions [22], [26], [27]. Most notable changes are achieved when LIF/serum conditions are replaced by serum-free 2i conditions, which efficiently block Erk signalling and GSK3 (glycogen synthase kinase 3) [25], [28], [29]. Cultured in 2i, mESCs exhibit lower expression of lineage-affiliated genes compared to LIF/serum conditions [25]. Moreover, the molecular heterogeneity with respect to Nanog and Rex1 is largely removed [30]. However, only recently it has been reported that also under LIF/serum conditions a more robust and homogeneous pluripotency state can be achieved [31]. It has been demonstrated that mESCs with a reduced Oct4 concentration express elevated and rather uniform levels of Nanog associated with an increased self-renewal capacity and delayed differentiation kinetics [31]. It has been suggested that the enhanced robustness of the pluripotency state under LIF/serum is facilitated by an increased sensitivity to LIF [31].

Taken together, all these observations indicate that extrinsic factors are critical determinants for the degree of mESC heterogeneity and differentiation. The cytokine FGF4 (fibroblast growth factor 4) is produced by undifferentiated cells in an autocrine manner [32] and initiates lineage commitment through Erk signalling [4], [33], [34]. Neither LIF nor serum can efficiently block the activation of Erk, suggesting that pluripotency is maintained by supressing commitment instructions downstream of this cascade [8], [28].

Considerable knowledge about signalling pathways and regulatory network structures governing mESC organization has accumulated over the last years. In particular, a couple of mechanisms associated with TF heterogeneity have been proposed quite recently [31], [35], [36], [37], [38]. However, details on their molecular basis and on the compatibility of these mechanisms are still missing. In many cases it remains unclear how extrinsic stimuli and cell-intrinsic (transcription) factors interact with each other to generate culture-dependent phenotypes. It is furthermore an unresolved question how and to what extent TF heterogeneity has a functional role in mESC pluripotency [34], [39]. In this work we apply a systems biological approach aiming on an embedding of different, diverse phenomena on mESC pluripotency and in the induction of differentiation within a consistent, quantitative description. Such reductionist's models allow delineating mechanistic concepts of biological function beyond intuitive reasoning and fostering the quantitative explanation of certain, potentially diverse phenomena. Specifically, we develop a mathematical model incorporating new experimental data using 2i conditions to explain the dynamic behaviour of mESCs in different cell states. With regard to theoretical findings on interactions between pluripotency factors such as Oct4, Sox2 and Nanog [40],[41] and stochastic effects in mESC decision making [20], [27], [42], [43], we specifically investigate a potential mechanism that integrates FGF4/Erk signalling into the proposed core network of pluripotency. This new regulatory component allows for an explicit modelling of the dynamic regulation of TF heterogeneity and for the induction of mESC differentiation.

Initially we set up a list of experimentally motivated criteria, which have to be consistently explained by our regulatory network model of mESC pluripotency:

- In **LIF/serum conditions** FGF4/Erk signalling is active and induces a heterogeneous, pluripotent stem cell state. This state is characterized by variable expression levels of Nanog (*criterion 1*) and Rex1 (*criterion 2*). The expression levels of both factors establish bimodal distributions defining different subpopulations [18], [22]. Simultaneous expression levels of Oct4 and Sox2 are constantly high and rather homogeneous (*criterion 3*) [34], [44].
- In contrast, **2i conditions** efficiently block FGF4/Erk signalling capturing mESCs in the pluripotent ground state. This cell state is characterized by high and homogeneous expression levels of all pluripotency factors, which establish unimodal peaked distributions (*criterion 4*) [26].
- Furthermore, under **LIF/serum** mESCs with low Nanog expression have an increased propensity for differentiation compared to cells with high Nanog expression (*criterion 5*) [18], [34].

To quantitatively compare our model results with experimental measurements and to validate the suggested mechanistic concept, we use a Rex1GFPd2 reporter cell line, in which a destabilized GFP protein is expressed from the Rex1 locus [45]. In contrast to the widely used Nanog-GFP reporter cell line [18], this construct ensures a comparable half-life of the GFP with Rex1 protein, which is essential to quantitatively monitor the dynamic behaviour of mESCs over relatively short time scales.

## Materials and Methods

### Network structure

**Figure 1:**
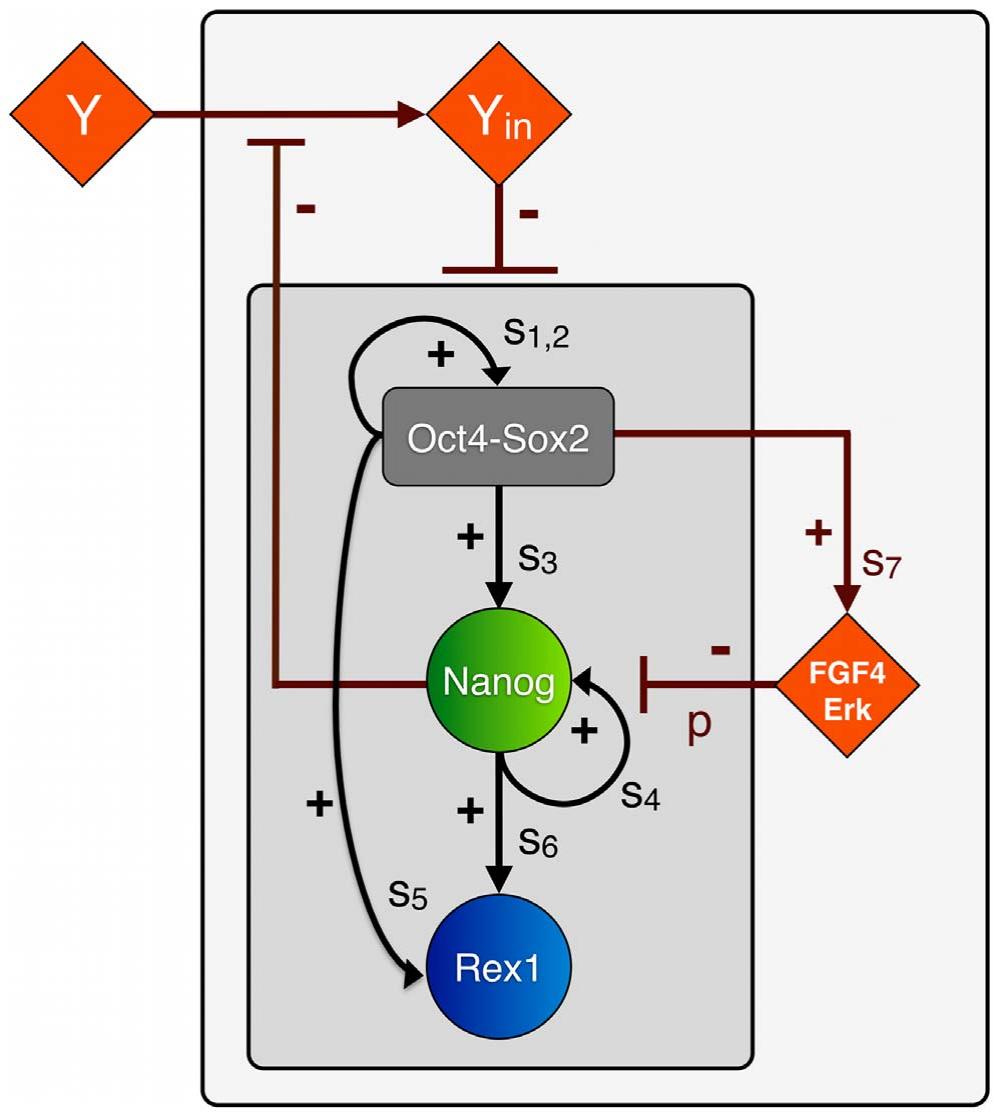
Model scheme of the regulatory network of mESC pluripotency. The core network (inner grey square) is composed of the TFs Oct4-Sox2, Nanog and Rex1, which are linked by positive feedback and feedforward loops (black arrows). The respective transcription rates are denoted by s_i_. The extended network (outer grey square) includes FGF4/Erk signalling and a differentiation signal Y, the latter facilitating the double-negative feedback loop from Nanog on all factors of the core network. FGF4/Erk is activated by Oct4-Sox2 and represses Nanog with rate p. High Nanog levels block the transmission of differentiation signal Y. The internal part of the differentiation cascade (denoted by Y_in_) negatively regulates the expression of Oct4-Sox2, Nanog and Rex1.

Relying on the principle of parsimony (in reference to “Occam's razor”) we restrict ourselves to the most simple model structure that consistently meets the criteria listed above. We consider the TFs Oct4, Sox2, Nanog and Rex1 as central elements of a self-regulating intracellular network structure (Figure 1, inner grey square). We assume that Oct4 and Sox2 proteins cooperate to positively regulate their own expression and to activate the transcription of Nanog and Rex1 to a basal level [6], [23], [24], [46]. In particular, we suppose that Oct4 and Sox2 form heterodimers before binding a certain promoter region. Additionally, we assume that the concentration of the heterodimers is in dynamic equilibrium with the steady state concentrations of Oct4 and Sox2 proteins. It is a reasonable simplification to solely account for the concentration of the heterodimers, instead of describing single protein concentrations [42]. Furthermore, we account for the finding that Nanog proteins form homodimers [47], [48], which establish an autoregulatory feedback loop [7], [35], [38] and motivate the choice of a Hill-coefficient n = 2 in the mathematical formulation below. In addition to the basal activation of Rex1 through Oct4 and Sox2, Nanog is considered to be an activator for the transcription of the pluripotency marker Rex1 [24]. Within the proposed model we explicitly describe the Rex1 dynamics to be able to compare simulation results with experimental data on the Rex1GFPd2 cell line. Rex1 is a sensitive marker for mESCs pluripotency and serves as a surrogate measure of Nanog expression.

Moreover, there is experimental evidence that Oct4 and Sox2 induce Erk activity through the activation of FGF4 and that Erk signalling acts as potential Nanog repressor [21], [49]. Hence, we include a negative, FGF4/Erk-mediated feedback loop into our network model (Figure 1, outer grey square).

In the first part of our study (comprising sections 1–3 in *Results*) we focus on the analysis of the negative feedback loop mediated by FGF/Erk signalling and how it affects the expression pattern of Nanog. Therefore, external factors such as differentiation signals are neglected in the first instance.

Recapitulating our findings on Nanog heterogeneity under LIF/serum conditions [42], we infer that Nanog levels critically regulate the transmission of differentiation signals and control the propensity for mESC differentiation (*gate-keeper function*). In the second part of our study (comprising sections 4 and 5 in *Results*) we quantitatively study this suggested mechanistic concept in the biological context of FGF4/Erk signalling. Therefore, we further amend the described network model by adding an indirect double-negative feedback loop from Nanog onto the pluripotency network (Figure 1). Taking Nanog overexpression studies into account [50], [51], we particularly propose that the transmission of a differentiation signal (or a signalling cascade) termed Y depends on the Nanog concentration of the cell. We assume that only sufficiently high Nanog levels can effectively block the (intracellular) propagation Y_in_ of the external signal Y. Y_in_ negatively regulates the concentrations of Oct4-Sox2, Nanog and Rex1. Specifically, we suppose that Y_in_ increases the degradation rates of the TFs. This assumption does not exclude the possibility that other negative regulations (e.g. transcriptional repression) are equally effective. As Oct4 is known to be essential for the maintenance of pluripotency and mESC self-renewal [6], [15], [52], we refer to the Oct4-Sox2-negative state as a differentiated cell state.

It should be emphasized that the proposed interactions do not necessarily represent direct regulations. In fact, our network edges are minimal representations summarizing presumable more complex and potentially indirect feedback loops. Especially, the proposed autoregulatory loops of Oct4-Sox2 and Nanog, as well as the signalling cascades including FGF4/Erk and Y may substitute for overall effects. In particular, new experimental findings indicate that Nanog directly regulates its own expression in a negative manner [35], [38]. However, the findings of Navarro et al. [35] and Fidalgo et al. [38] do not rule out that additional, intertwined positive feedbacks loops (established by cofactors like Klf4 or Esrrb) are dominantly present and required to maintain mESCs pluripotency. Therefore, we remain with the assumption of an overall positive, autoregulative feedback of Nanog and discuss the impact of an additional negative feedback regulation later in the *Discussion*.

A related model approach has been applied by Chickarmane et al. [43], who extended previously established network models [41], [42] by FGF4/GSK3 signalling and an intracellular differentiation gene termed G. In contrast to the external, culture-dependent differentiation signal Y analysed in our approach, G is an intrinsic factor, which is regulated by the mESC circuit itself. We will later discuss the differences between these two alternative model approaches with respect to mESC differentiation.

## Mathematical Model

For the quantitative assessment of the model structure, we derive a mathematical description of the proposed interactions between the TFs Oct4, Sox2, Nanog, Rex1 and the signalling pathways FGF4/Erk and Y (cf. Figure 1). In particular, the interaction dynamics between Oct4-Sox2, Nanog, Rex1 and FGF4/Erk are described in terms of their intracellular protein concentrations, which are denoted by [OS], [N], [R] and [E]. For reasons of simplicity, these interactions are solely described on the transcriptional level. That means, we intentionally neglect other (e.g. post-transcriptional) regulatory effects and time delays and consider that the transcription of a gene ultimately results in the production of the corresponding protein. The temporal changes of the protein concentrations [OS], [N], [R] and [E] are represented by the following set of coupled stochastic differential equations:
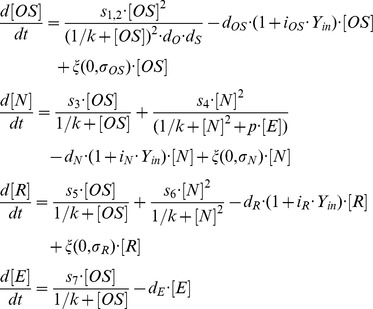



We describe the transcriptional regulation of all network factors using Hill kinetics, i.e. we define parameters such as transcription rates s_i_ (with i ∈ (1,2,…,7)) and a binding rate k to model molecular processes in single mESCs. The regulation of the Oct4-Sox2 heterodimer is described by a combined transcription rate termed s_1,2_, which is composed of two transcription rates s_1_ and s_2_ for Oct4 and Sox2 respectively and a formation rate (cf. [42]). The repression rate of Nanog is denoted by p. All proteins and protein complexes are degraded by first-order kinetics with protein specific degradation rates d_j_ (with j ∈ (OS, N, R, E)). These degradation rates are enhanced by inhibition factors i_j_ depending on the intracellular activity of a differentiation signal Y, denoted by Y_in_. Y_in_ is implemented as S-shaped function of the Nanog concentration [N] and signal Y:
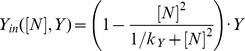



As stated in the previous paragraph, we assume that Y is an external, culture-dependent signal. The respective implementation has been chosen to ensure the following criteria: (1) in the absence of Nanog, the maximum level of Y_in_ is Y, and (2) if the Nanog level is low, Y_in_ approaches zero. Binding rate k_Y_ changes the slope of the S-shaped curve, but does not alter the minimum and maximum level of Y_in_.

Furthermore, the expression dynamics of the TFs Oct4-Sox2, Nanog and Rex1 are affected by a transcriptional background noise. In particular, the concentrations [OS], [N] and [R] contain a stochastic part termed ξ, which represents an approximation of multiple sources of noise that might occur on the molecular level. The stochastic part is implemented as zero-mean Gaussian process, which is multiplied by the respective protein concentration. The parameter σ_j_ defines the TF-specific noise amplitude. The multiplicative formulation is chosen because the variability of the protein levels within the low expressing state (i.e. the width of the low peak) is much smaller compared to the variability in the high expressing state (i.e. the width of the high peak). Given the abundance of detected protein levels, (additive) stochastic fluctuations due to small molecule numbers can hardly account for the observations. On the logarithmic scale for the fluorescence levels the distributions appear almost equally wide, arguing in favour of the multiplicative approach. Negative protein concentrations are excluded by setting the lower bound for all concentrations equal to zero. For reasons of simplicity, we do not consider random fluctuations on the signalling pathways FGF4/Erk and Y.

### Model Parameters

**Figure 2:**
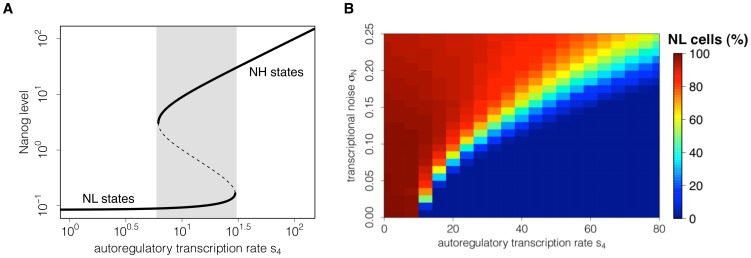
Dependency of stable Nanog states on Nanog autoregulation and noise. (A) The bifurcation diagram indicates the existence of Nanog states depending on the autoregulatory transcription rate s_4_. The lower solid line shows the existence of Nanog-low (NL) states and the upper solid line shows the existence of Nanog-high (NH) states. Within the bistable region (shaded in grey) coexisting stable states are separated by unstable states (dashed line). (B) Simulating a cell population, the heat map illustrates the proportion of NL cells depending on the transcription rate s_4_ and on the transcriptional noise σ_N_ at time point t = 4320min (i.e. 3 days of in silico culture). For any value of the background noise, an increase in the transcription rate s_4_ reduces the proportion of NL cells.

Depending on the choice of the model parameters, the system (here a single mESC) can reach different equilibrium states, formally denoted as *stable steady states* or *attractor states*. In the context of biological regulatory networks, different attractors states are often considered to represent different cell fates or developmental states [12], [53], [54]. Since we assume that the underlying network structure is the same for all cells regardless of specific culture conditions, the number of available attractor states is solely determined by the intensity of the network interactions (i.e. by the model parameters). To identify critical parameters that qualitatively change the expression pattern of Nanog, we perform stability and bifurcation analysis using the software tool xppaut. Additionally we performed simulation studies on the dependency of TF distributions on parameter variations (cf. Figure 2 and Figure S3–S4 in File S1). Based on these simulation studies, we identified a set of model parameters, which allows reproducing the experimentally observed TF distributions under 2i and LIF/serum conditions [18], [20], [22], [25] with minimal changes (cf. *Results*). Further details on the model parameters can be found in File S1.

### Simulation procedure

The Euler-Maruyama method has been applied to approximate numerical solutions of the stochastic differential equations. The simulations have been implemented using the programming language C. The source codes will be provided by the authors upon request. Data analysis and images were conducted using the statistic software R (http://www.r-project.org/). Further details on the simulation procedure can be found in File S1.

### Mouse embryonic stem cell culture

Rex1GFPd2 embryonic stem cells (described in [45]) were cultured without feeders on plastic coated with 0.1% gelatine either in LIF/serum conditions (GMEM (Sigma, cat. G5154) supplemented with 10% FCS (Sigma, cat. F7524), 100 mM 2-mercaptoethanol (Sigma, cat. M7522), 13 MEM nonessential amino acids (Invitrogen, cat. 1140-036), 2 mM L-glutamine, 1 mM sodium pyruvate (both from Invitrogen), and 100 units/ml LIF), or in the serum-free media N2B27 (NDiff N2B27 base medium, Stem Cell Sciences Ltd, cat. SCS-SF-NB-02) supplemented with small-molecule inhibitors PD (1 mM, PD0325901) and CH (3 mM, CHIR99021) as described [28].

### Flow Cytometry

After treatment with Accutase, live mESCs were resuspended in PBS with 1% FCS and ToPro-3 (Invitrogen) was added at a concentration of 0.05 nM to detect dead cells. Flow cytometry analyses were performed using a Dako Cytomation CyAn ADP high-performance cytometer. Data was analysed using FlowJo software. The data will be provided by the authors upon request.

### Gene Expression Analysis by Quantitative PCR with Reverse Transcription

Rex1GFPd2 cells were plated in 2i medium at a density of 1.5×10e4 cells/cm2. 24 hours after plating, 2i medium was replaced with N2B27. Cells were harvested at every 3 hours after media change and total RNA was extracted using RNeasy kit (Qiagen). cDNA was synthesized with SuperScript II RT (Invitrogen) was subjected to quantitative PCR using Taqman probe system (Applied Biosystems). The data will be provided by the authors upon request.

## Results

### Stability analysis of Nanog

The autoregulation of Nanog establishes a positive feedback loop, which leads to distinct stable steady states. For the case that the autoregulative capacity of Nanog is rather weak (i.e. for a low transcription rate s_4_), Nanog concentration is not sufficient to stimulate its own transcription. As result, Nanog transcription is solely driven by activation through Oct4-Sox2 and remains at rather low levels (Nanog-low state - NL, lower solid line in Figure 2A). In contrast, if the transcription rate s_4_ is high, Nanog acts as a potent enhancer of its own transcription and sustains the autoactivation cycle. Thus, a second stable steady state at high Nanog levels is established (Nanog-high state – NH, indicated by the upper solid line in Figure 2A). Although the NL states only exist for low values of rate s_4_ and the NH state is restricted to strong autoactivation, there is an intermediate region (indicated in grey in Figure 2A), in which both states coexist simultaneously. For any value of the rate s_4_ within this range a single mESC can attain either of the two Nanog states (bistability).

For a purely deterministic system (i.e. a system that does not account for any stochastic effects), the choice of either of the two stable Nanog states solely depends on the rate s_4_ and the initial TF concentrations. However, it has been demonstrated experimentally that mESCs can switch between NH and NL states [18], [20]. We have previously shown that, in a bistable system, a transcriptional background noise is capable to induce reversibly changing expression levels [42]. Thus, we assume that the expression levels of Nanog are affected by random fluctuations (noise) arising from the stochastic nature of underlying molecular processes and chemical reactions (cf. Materials and Methods). Due to this unspecific stochastic component, Nanog concentrations no longer approach (singular) stable steady states, but rather reside in or fluctuate between so-called attractor basins. These basins correspond to the vicinity of stable steady states that are given by the deterministic network.

While the autoregulatory transcription rate s_4_ regulates the availability of stable Nanog states (Figure 2A), in a bistable system, the intensity of the transcriptional noise (denoted by σ_N_) _determines_ the frequency of state transitions. It critically regulates the proportion of cells in the NH and NL state. Figure 2B illustrates the dependency of the NL population on the transcription rate s_4_ and the noise intensity. For the case that the autoregulative capacity of Nanog is weak (s_4_<20), even small perturbations (σ_N_<0.1) are sufficient to induce a large fraction of NL cells (red colour). In contrast, if the autoregulative capacity of Nanog is high (s_4_>60), cells with high Nanog expression levels (blue colour) are prevailing and only strong perturbations (σ_N_>0.2) lead to the establishment of a NL fraction. Moderate intensities of both rates allow for a dynamic equilibrium with different intermediate cell fractions (light blue to yellow colour). The heat map in Figure 2B furthermore demonstrates that an increase in the transcription rate s_4_ reduces the fraction of NL cells for any value of the transcriptional background noise (horizontal transition from red to blue colour).

In the following we analyse how these theoretical findings can be explained in the biological context of FGF4/Erk signalling and ground state mESCs. Specifically, we investigate whether different intensities of the FGF4/Erk-mediated feedback loop lead to different expression patterns of Nanog, and whether these patterns can be mapped onto the phenotypic differences between LIF/serum and 2i conditions (cf. *Introduction*, criterion 1–5).

### FGF4/Erk signalling regulates expression patterns of self-renewing mESCs

**Figure 3:**
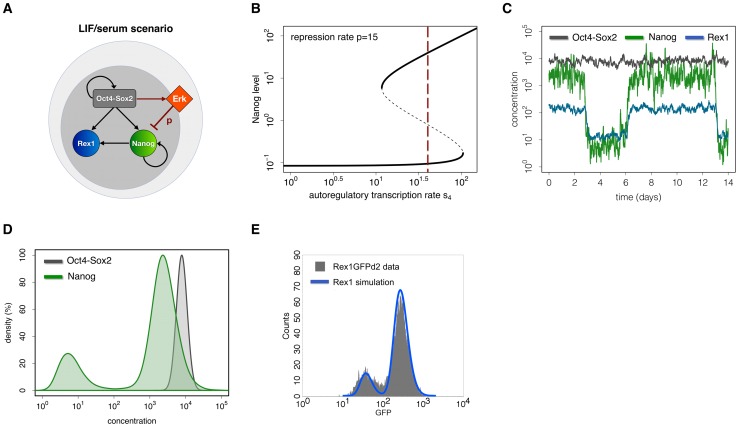
Mechanistic explanation and simulation results for the LIF/serum scenario. (A) Model scheme. Autocrine FGF4/Erk signalling is proposed to inhibit the transcription of Nanog at rate p. In the LIF/serum scenario Erk signalling is active (i.e. p>0). (B) Bifurcation diagram. Assuming a constant transcription rate s4 (vertical red line) under LIF/serum mESCs are captured in a bistable region (p = 15) with respect to Nanog expression. (C) Single cell trajectories. The diagram shows simulated trajectories of Oct4-Sox2 (grey), Nanog (green) and Rex1 (blue) concentrations for the LIF/serum scenario. (D) Simulated TF distributions of Nanog (green) and Oct4-Sox2 (grey) within mESC populations at time point t = 4320 min (i.e. 3 days of in silico culture). In the LIF/serum scenario Nanog is subject to state changes establishing a bimodal TF distribution. The curves are normalized to match the local maxima for high expression states under LIF/serum conditions. (E) Comparison of the simulation result for Rex1 (blue line) with experimental data (grey histogram) obtained from flow cytometry analysis of Rex1GFPd2 mESCs maintained in LIF/serum. The parameter set used for these simulations is given in Table S1 in File S1.

For a conceptual understanding of the nature of mESC culture conditions, one can distinguish two model scenarios. The first scenario, mimicking LIF/serum conditions, is characterized by activated Erk signalling (e.g. through FGF4 and serum factors). Erk acts as an inhibitor of Nanog transcription (i.e. the repression rate p is greater than zero, Figure 3A). As demonstrated in the previous section, the autoregulative Nanog transcription rate s_4_ can be adjusted such that the NH and the NL state exist simultaneously (Figure 3B choice of s_4_ is indicated by the red line), while a moderate background noise σ_N_ can induce reversible state transitions.

In order to meet criterion 1 (cf. *Introduction*), the repression rate p, the transcription rate s_4_ and the Nanog-specific noise intensity σ_N_ are adjusted such that mESCs are able to switch between the NH and the NL state (green line in Figure 3C and Figure S1 in File S1). Nanog expression levels therefore establish a bimodal distribution (green distribution in Figure 3D) as observed experimentally [18], [20]. Moreover, the proportion of the two subpopulations and the difference in their Nanog expression levels, which are regulated by the transcription rates s_3_, s_4_ and the noise intensity σ_N_, are adapted according to these experimental findings (i.e. with around 20% NL, 80% NH cells and a difference of two log scales, cf. File S1). In addition to its autoregulatory capacity, Nanog activates the transcription of Rex1. Rex1 serves as an experimentally accessible readout reflecting cellular Nanog concentrations. We found that the regulatory rates of Rex1 are critical for the establishment of two Rex1 subpopulations. If the rates of Rex1 (i.e. the transcription and degradation rates) are high, changes in the Nanog concentrations are instantaneously transmitted to Rex1 (blue line in Figure 3C). Thus, a bimodal distribution of Rex1-high (RH) and Rex1-low (RL) cells is established (criterion 2, blue distribution in Figure 3E). However, if the regulatory rates are low, the turnover of Rex1 protein is reduced and changes in the concentration of Nanog will only slowly effect the Rex1 expression. Consequently, the Rex1 concentration would range at an intermediate level between the RH and the RL peak while a unimodal distribution is established. The transcription rates s_5_ and s_6_ are adjusted to fit the distribution of Rex1GFPd2 mESCs measured by flow cytometry (grey histogram in Figure 3E). Furthermore, the combined transcription rate s_1,2_ and the Oct4-Sox2-specific noise strength *σ_OS_* are adapted such that the concentrations of the heterodimer remain constantly high (grey line in Figure 3C) and are rather homogeneously distributed (criterion 3, grey distribution in Figure 3D).

**Figure 4:**
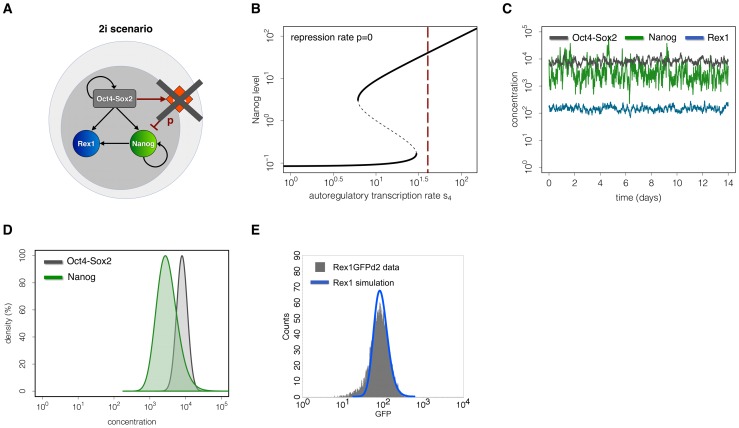
Mechanistic explanation and simulation results for the 2i scenario. (A) Model scheme. In the 2i scenario Erk signalling is efficiently blocked (i.e. p = 0). (B) Bifurcation diagram. Keeping all regulatory rates and the transcriptional noise constant, the removal of the Erk repression in 2i shifts the Nanog level into a monostable region. (C) Single cell trajectories. The diagram shows simulated trajectories of Oct4-Sox2 (grey), Nanog (green) and Rex1 (blue) concentrations for the 2i scenario. (D) Simulated TF distributions of Nanog (green) and Oct4-Sox2 (grey) within mESC populations at time point t = 4320 (i.e. 3 days of in silico culture). The homogenous high expression levels under 2i result in unimodal TF distributions. (E) Comparison of the simulation result for Rex1 (blue line) with experimental data (grey histogram) obtained from flow cytometry analysis of Rex1GFPd2 mESCs maintained in 2i. The parameter set for these simulations is given in Table S1 in File S1.

In the second scenario, mimicking 2i conditions, Erk signalling is blocked very efficiently. Thus the negative regulation on Nanog transcription is removed (i.e. the repression rate p is equal to zero, Figure 4A). Keeping all other parameters fixed, the removal of the repression rate p leads to a shift of the systems dynamics into a monostable regime, i.e. only the NH state remains (Figure 4B, intersection with the red line). In such a monostable setting, perturbations (e.g. due to transcriptional noise) have no regulatory effect and the system is trapped in the vicinity of the unique stable steady state. As demonstrated by simulated time courses of TF expression levels (Figure 4C), the inhibition of Erk signalling accounts for rather high and homogeneous levels of Nanog and Rex1 and for the establishment of unimodal, peaked distributions as required to meet criterion 4 (Figure 4D and 4E). Since Oct4-Sox2 concentrations are unaffected by repressive FGF4/Erk signalling, expression levels of these TFs remain unchanged compared to the LIF/serum scenario. Here, we emphasize that the simulated TF distributions in the 2i scenario directly result from the parameter set used for the LIF/serum scenario except that the repression rate p is equal to zero. All other parameters, especially the autoregulatory rate s_4_ and the transcriptional background noise, remain unchanged.

These results demonstrate that the impairment of the FGF4/Erk-mediated Nanog suppression under 2i is sufficient to change the cellular state compared to LIF/serum conditions.

### State transitions of mESCs under LIF/serum

In terms of the proposed interaction network, state transitions have been defined as stochastic switches from one attractor basin (the NH or NL basin) into the other one. Formally, we require that a cell resides in the opposing attractor state for a certain time period (i.e. for more than one hour) to accept the transition as valid. Thus, we are able to distinguish substantial changes in the cell's expression pattern from stochastic fluctuations.

In the 2i model scenario only the NH expression pattern is supported. Hence, stochastic state transitions between the different Nanog expression states are *per se* not possible. In contrast, the LIF/serum scenario allows for the existence of a second expression pattern at lower Nanog levels. State transitions between the NH and the NL basin can occur. However, according to our simulation results even in the LIF/serum scenario state transitions are predicted to be rare events. Analysing simulated single cell trajectories (cf. Figure S1 in File S1) we estimate a number of 0.05 transitions per cell per 24-hour interval (or alternatively 0.0021 state transitions per hour). That means only 5 out of 100 cells are expected to change their expression state within 24 hours. Furthermore, we found that for constant transition probabilities, simulated residence times of mESCs in the NH and the NL state approach an exponential distribution with mean residence time of around 9 days for the NL state and around 11 days for the NH state (Figure S2 in File S1). However, for constant interaction rates the frequency of state transitions and consequently the mean residence times are mainly determined by the transcriptional background noise σ_N_.

**Figure 5:**
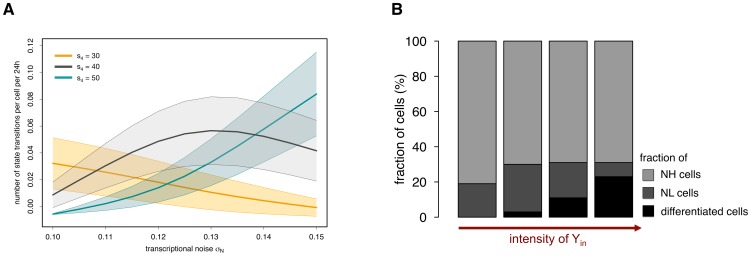
Rate changes in the LIF/serum scenario. (A) The number of state transitions depends on the background noise σ_N_. In the bistable region, for any value of the autoregulatory transcription rate s_4_ (e.g. orange line: s_4_ = 30; grey line: s_4_ = 40; blue line: s_4_ = 50), an increase in the noise intensity σ_N_ up to a certain range leads to an increase in the number of state transitions. If the intensity of the background noise becomes too high, it interferes with the underlying TF dynamics and the number of valid transitions decreases. Thick middle lines show the mean number of state transitions in 24 h for the respective value of s_4_, upper and lower bands depict the standard deviations. (B) Simulation results demonstrate that the fraction of terminally differentiated cells depends on Y_in_, which is determined by the culture conditions (i.e. intensity of signal Y) and by the Nanog concentration. Cell fractions are determined after simulating 3 days of in silico culture with the parameter set for the LIF/serum scenario.

Figure 5A illustrates the expected number of state transitions per cell per 24-hour interval depending on the noise intensity σ_N_ and for different values of the autoregulatory transcription rate s_4_. It becomes clear that for an intermediate rate s_4_ (shown in grey), a higher background noise σ_N_ forces mESCs to change their expression state more often, which leads to an initial increase in the number of transitions. However, if the background noise becomes predominant (i.e. σ_N_>0.14), it loses its regulatory function and simply overlays the underlying TF dynamics, i.e. mESCs can no longer reside in the attractor basins (the NH or the NL one). This results in a decrease of valid state transitions. This behaviour can be observed for any value of the transcription rate s_4_ within the bistable region. However, to achieve a comparable number of state transitions the noise intensity σ_N_ has to be higher for higher values of s_4_.

It has been shown experimentally that NH and NL subpopulations have distinct properties with NL cells being more prone to differentiation [18], [21]. For that reason, we evaluate our model predictions with respect to Nanog-related interaction rates that can alter the proportion of NL cells in a mESC population maintained under LIF/serum conditions. As demonstrated in Figure 2B, the fraction of NL cells can be shifted either by reducing intrinsic perturbations (i.e. the transcriptional noise) or by manipulating the underlying system dynamics (e.g. by reducing FGF4/Erk-mediated Nanog repression, cf. Figure 3B and 4B). However, our model also predicts that an increase in Nanog expression (e.g. by altering the autoregulatory transcription rate or the input rate from Oct4-Sox2) reduces the fraction of NL cells, such that a more homogeneous expression pattern can be established (Figure S4–S5 in File S1). This statement is even true in the presence of FGF4/Erk signalling. Taking Nanog overexpression studies into consideration [50], [51], these findings are in a first instance not surprising. However, in addition to evaluating model predictions on the population level, our model approach also allows to study the effect of rate changes on the underlying system dynamics. In particular, if Nanog is elevated through an increase of its own autoregulatory capacity, the expression pattern of Nanog is shifted towards the monostable NH regime. In this regime, FGF4/Erk signalling is simply less effective given the higher Nanog activation. However, if the Oct4-Sox2-mediated input rate becomes more potent, the concentration in the NL state approaches the NH concentration, which also leads to a reduction of NL cells and a more uniform Nanog distribution (Figure S5 in File S1). These predictions are consistent with experimental findings, which demonstrate that cellular mESC states are interchangeable [25] and that homogeneous expression patterns of Nanog (i.e. a “2i-like” state) can be achieved under LIF/serum conditions [31].

### Nanog retains pluripotency in LIF/serum conditions

It has been demonstrated that mESCs with low Nanog expression have a high tendency to differentiate [18], [20]. Therefore, we investigate particular mechanisms that are suited to translate the TF heterogeneity into a functional regulation of mESC self-renewal and differentiation. One mechanistic explanation for this phenomenon is shown in Figure 1. In particular, we assume that Nanog concentrations can critically regulate the transmission of differentiation signals [8], [34], which are modelled by an extrinsic, culture-dependent factor Y. The integration of a double negative feedback loop from Nanog onto the pluripotency factors Oct4-Sox2, Nanog and Rex1 naturally entails stochastic differentiation events under LIF/serum conditions.

In the previous section, we have demonstrated that in LIF/serum mESCs are subject to occasional state changes. The proportion of NL cells and, therefore, the number of cells susceptible to differentiation signal Y, is critically regulated by the transcription rate s_4_ and the noise intensity σ_N_ (Figure 2B). The fraction of NL cells that actually differentiates depends on the intracellular activity of signal Y (referred to as Y_in_). Y_in_ depends, by definition (cf. Materials and Methods), on the Nanog expression of the cell and on the strength of signal Y. If signal Y is low, Y_in_ is also low and its repressive activity is not sufficient to downregulate Oct4-Sox2 expression, even if mESCs are in the NL state. That means all NL cells eventually re-express Nanog and no differentiation event occurs (first bar in Figure 5B). If signal Y is sufficiently strong, also the repression by Y_in_ is strong and cells in the NL state can differentiate (last three bars in Figure 5B). However, we found that for an intermediate range of Y and within a stated time interval (e.g. 3 days) some mESCs re-express Nanog while others differentiate by chance. It should be pointed out that the precise amount of terminally differentiated cells is not predictable with our type of model description, since cellular processes like proliferation and cell death, which can alter these fractions decisively, are currently neglected.

In contrast to LIF/serum conditions, 2i conditions abrogate pro-differentiation activities and thus inhibit the induction of differentiation. However, the question whether the observed variability of Nanog under LIF/serum evolves as part of a general regulatory mechanism, which is required for mESC differentiation, or whether it represents an artefact induced by the culture conditions (e.g. by serum factors) remains. In the last part of our model-based analysis we investigate the dynamics of state transitions as they occur at the onset of mESC differentiation.

### Differentiation dynamics

**Figure 6:**
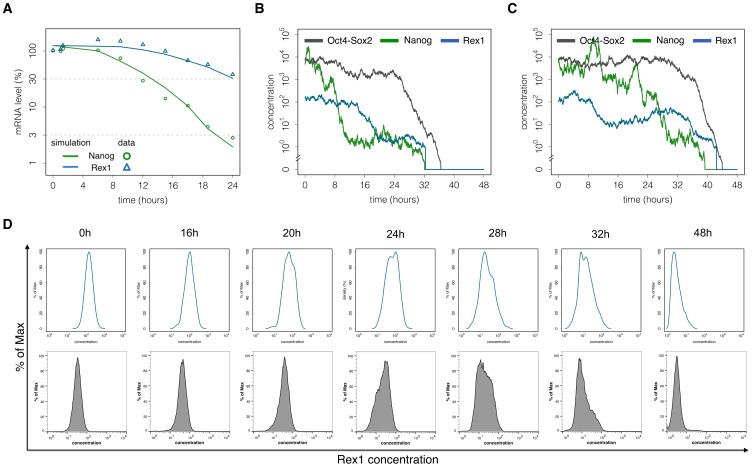
The differentiation process of mESCs. (A) Experimental and simulated TF kinetics. mRNA levels of Nanog (green circles) and Rex1 (blue triangles) are measured after 2i withdrawal and used to adapt the velocity of the differentiation process in terms of the proposed network model. Solid lines depict the respective simulation results of average Nanog and Rex1 levels. (B) – (C) Single cell trajectories. The Nanog level (green) initially switches from the NH into the NL state. Rex1 expression levels gradually decrease (blue). The Oct4-Sox2 level (black) is suddenly downregulated if the intracellular activity Y_in_ becomes sufficiently strong. (D) Flow cytometry measurements of Rex1GFPd2 mESCs show intercellular differences in the differentiation dynamics of Rex1 (lower panel). The respective model results are consistent with the experimental findings (upper panel). The parameter set for these simulations is given in Table S2 in File S1.

2i conditions promote homogenous undifferentiated mESC populations, thus providing an appropriate system to study the dynamics of mESCs upon initiation of differentiation. This is achieved by removing the two inhibitor molecules from the serum-free N2B27 basal medium [55]. Measurements of Nanog and Rex1 mRNA levels at defined time points after the removal of inhibitors (Figure 6A) show that both pluripotency factors are downregulated, although the kinetics differ. In particular, Nanog levels rapidly decline while Rex1 initially persists at high levels before decreasing. These kinetics are consistent with recent findings by MacArthur et al. [36] demonstrating that the loss of pluripotency occurs on a longer timescale compared to the loss of Nanog.

In terms of our network model, we propose that the removal of the two inhibitors causes an accumulation of Erk. This is modelled by a time-dependent, stochastic process increasing the repression rate p (cf. File S1). The stochastic part models natural cell-to-cell variations in the transduction of autocrine FGF4 signalling, e.g. due to differences in their spatial arrangement or in local cell densities. The time-dependency of rate p is estimated from qRT-PCR measurements (points and triangles in Figure 6A). In particular, we initially fitted the increase of rate p to the Nanog time course (green line in Figure 6A). We found that for a consistent description of the Rex1 kinetics (blue line in Figure 6A), the turnover of Rex1 has to be reduced compared to LIF/serum conditions. Furthermore, we examine simulated single cell trajectories. Two examples are shown in Figure 6B and 6C. They demonstrate that the Nanog expression level (green line) initially switches from the NH into the NL state, followed by a gradual decrease of the Rex1 level (blue line). As we furthermore assume that N2B27 conditions promote differentiation signals, which are transmitted in case of low Nanog levels (as discussed in the previous section), we obtain a terminal downregulation of Oct4-Sox2 expression levels (black line) and the acquisition of a differentiated cell state. Although the mechanism driving mESC differentiation is identical in all cells, TF trajectories indicate differences in the differentiation dynamics. Especially between 8 h–20 h after the induction of differentiation the model predicts a great variability in the expression levels of Nanog and later, between 20 h–32 h, in the expression of Rex1 (cf. Figure S6 in File S1), consistent with recent experimental observations [45], [56] and our own results (TK, data unpublished).

Since differences in the downregulation of TFs in individual cells are not detectable by qRT-PCR measurements of populations, flow cytometry is an appropriate technique to address the intercellular variability of differentiation dynamics. We compare our model predictions (upper panel in Figure 6D) to biological data on Rex1 expression obtained by flow cytometry at defined time points after the induction of differentiation using a Rex1GFPd2 mESC line (lower panel in Figure 6D). The resulting data reveal an asynchronous differentiation process with an intermediate, heterogeneous period in which Rex1 levels differ significantly between cells. Starting with a homogeneous mESC population with respect to Rex1 expression levels (0 h–20 h), the distribution becomes wider between 20 to 32 hours after 2i withdrawal indicating differences in the velocity of the Rex1 downregulation. 48 h after the induction of differentiation most of the cells contain no or only low levels of Rex1, although a minority retain rather high expression levels. Our model results are in very good agreement with the experimentally observed dynamic behaviour of Rex1GFPd2 after 2i withdrawal (upper panel in Figure 6D and Figure S6 in File S1).

## Discussion

We have established a new mathematical description of molecular regulation in mESCs. Extending our previously published model of the regulatory circuit between Oct4, Sox2 and Nanog, we here integrated Rex1 expression as well as FGF4/Erk signalling to achieve a more detailed and quantitative understanding on how specific culture conditions influence transcription factor expression and generate experimentally observed phenotypes.

We demonstrated that transcriptional Nanog repression by FGF4/Erk signalling is a suitable candidate mechanism to transfer mESCs from a pluripotent ground state, in which only a Nanog high state is permitted, into a cellular state, in which a Nanog high (NH) and a Nanog low (NL) regulation pattern simultaneously coexist (bistability). Transcriptional fluctuations can trigger transitions between these two Nanog patterns. Evaluating the LIF/serum model scenario with respect to the frequency of transitions, we conclude from our model results that (in a limited period of time) observable state transitions are erratic and rare events. Moreover, we expect prolonged residence times in the order of several days for both Nanog expression states, which might in fact exceed typical cell cycle times or lifespans of individual mESCs. This could be a potential reason why experimental evidence for reversible state transitions on a single cell level is still limited. Nevertheless, stochastic fluctuations emerge as a critical factor in the regulation of the probability of state transitions. Enhanced intrinsic fluctuations, which might be caused by external factors or stress, elevate the number of state transitions and thus reduce residence times respectively.

Others and we have previously commented on the different propensities of NH vs NL cells to respond to differentiation inducing conditions. In order to further elucidate this aspect and to make our model comparable to experimental findings, we included Rex1 as a reliable marker of an undifferentiated cell state in our network. In particular, we assumed Rex1 to be a direct target of Nanog, thus reflecting the prevailing Nanog concentration. With this model extension we are able to consistently account for the establishment of Rex1 subpopulations under LIF/serum conditions [22], [25]. Furthermore, we studied the role of extrinsic, culture-dependent differentiation promoting signals, which we summarize into the model variable Y. Putting the transmission of signal Y under the governance of Nanog, we are able to consistently reproduce key features of pluripotency regulation. Although we have suggested this mechanistic concept previously, we here provide a quantitative study on its implications in the context of heterogeneous LIF/serum conditions and in the differentiation process initiated by 2i removal. Based on our network model, the decision process between Nanog-regain and differentiation is stochastic. However, in cell cultures other parameters like local cell densities and cell-cell interactions might effect the distribution and accessibility of external signalling molecules (e.g. cytokines, protein kinases, small molecules). Therefore, the assumption of a purely stochastic mechanism should be further evaluated with respect to potential correlation between the environment of a cell and its fate.

We also applied the extended model of mESCs regulation to study TF dynamics at the onset of differentiation. The outlined model consistently explains individual cell differences in the dynamics of the differentiation process initiated by removal of the 2i inhibitors. In particular, we showed that an asynchronous differentiation process with an intermediate, heterogeneous period of Rex1 expression can result directly from variability in the individual cellular responses to the inhibitor removal. Furthermore, we are able to predict single cell kinetics, which might underlie the experimentally observed population behaviour. Although the correspondence of data and model suggest a stepwise process accounting for the changing Nanog expression, further measurements on the temporal dynamics of Nanog expression in individual cells are required to stringently rule out a continuous or even linear process.

Karwacki-Neisius et al. [31] recently reported on the establishment of a more robust pluripotency state under LIF/serum by narrowing the range of Oct4 expression levels in mESCs. They demonstrate that Oct4^+/−^ mESCs exhibit elevated levels of Nanog due to a reduction of the proportion of NL cells [31]. In our network model, Oct4-Sox2 heterodimer induce Erk signalling through the activation of FGF4. Thus, a reduction of Oct4 would lead to a reduction of FGF4/Erk signalling and consequently to a more homogeneous expression of Nanog associated with a higher self-renewing capacity. However, in contrast to the experimental findings of Karwacki-Neisius et al. [31], we would expect reduced FGF4 levels if Oct4 expression is lowered. The experimentally observed inability of the cells to respond appropriately to FGF signalling, together with a higher sensitivity to LIF indicates a more complex and most likely concentration-dependent function of Oct4 [31]. This level of complexity exceeds our current model description. However, our simple network model is consistent with the experimental findings on mESC differentiation presented in this study. Karwacki-Neisius et al. [31] intensively studied differentiation kinetics of Oct4^+/−^ mESCs and demonstrated that the downregulation of pluripotency factors such as Rex1, Sox2 or Esrrb is delayed in these cells. Since Oct4-low cells can only emerge from the NL population, they conclude that the differentiation impairment results from the lack of NL cells. These findings are consistent with our model perspective describing mESC differentiation as a two step process, in which only primed mESCs in the NL state are susceptible to external differentiation signals (*gate-keeper function* of Nanog).

Our model complements a recent mESC model presented by Chickarmane et al. [43], in which stochastic cell fates are caused by mutual antagonism between Nanog and a lineage-affiliated TF (termed gene G) in conjunction with internal noise. In contrast to our model approach, in which differentiation cues are provided and regulated by the cell's environment (i.e. by the culture conditions), the alternative model suggests spontaneous differentiation events that are triggered by the mESC circuitry itself [43]. Thus, mESC differentiation becomes independent of the culture conditions and stochastic switches to a differentiated cell state can occur even for very low Nanog levels. However, the fact that Nanog-null mESCs can be maintained under 2i culture conditions [21] without differentiation, contradicts this assumption and hints towards external effectors regulating mESC differentiation by culture dependent signals as considered here.

The precise nature of differentiation inducing signals is not yet resolved. Several candidates, e.g. FGF, Wnt or Notch signalling, show the required functionality (i.e. the potential to induce differentiation), but it is not clear that signal Y might correspond to just one particular mechanism. In fact, there might be a plethora of potentially interacting pathways and signals that generate the Nanog-depend activity under LIF/serum conditions. Moreover, recent findings demonstrate that the gene regulatory network of mESCs is highly flexible with overlapping functional activities between TFs and signalling pathways [56], [57]. These studies indicate that Nanog is only one part in a rather complex, mechanistic setup protecting mESCs from differentiation [30]. Therefore, the impact of redundancy, especially on the dynamics of differentiation, has to be further explored.

We are aware that the predictive power of our modelling approach is determined by the set of underlying assumptions. In particular, in the outlined model the positive feedback regulation of Nanog is essential to generate the bistable expression pattern in LIF/serum. However, this feedback mechanism is not restricted to an autoregulation of Nanog itself, but can also result from a cooperative activation by known (co-)factors like Esrrb, Klf4 of FoxD3 [56], [57], [58], [59], [60]. For our studies, which focus on the effect of FGF4/Erk signalling, we have summarized all the potential sources for Nanog activation by a single autoregulatory loop. This simplification might be a limiting factor in studies that examine other parts of the pluripotency network. However, the model network can be extended by additional (intermediate) factors without destroying the underlying bistability. Navarro et al. [35] and Fidalgo et al. [38] recently demonstrate that Nanog autorepression plays an important role in the regulation of Nanog heterogeneity. Although our model network does currently not incorporate a negative Nanog autoregulation, we can speculate about the effect of an additional negative feedback regulation. We argue that the integration of Nanog autorepression would lead to a reduction of the Nanog expression level in the NH state, but does not inevitably change the system dynamics with respect to heterogeneous expression patterns under LIF/serum, as long as the repression is moderate. If Nanog autorepression becomes predominant, the NH state vanishes and all mESCs gradually differentiate under LIF/serum conditions due to the loss of protective Nanog. However, to be able to quantitatively study the effect of Nanog autorepression the network model has to be modified accordingly.

In conclusion, although our modelling approach is rather simplistic and has, therefore, a number of limitations, it clearly demonstrates that the determination of the mESC state by the strength of a negative feedback loop mediated by FGF4/Erk signalling, which itself is controlled by the culture conditions, would be a consistent explanation of the experimental findings. Within this context, silencing of the FGF4/Erk-mediated feedback generates a unique cellular state, in which only high expression levels of pluripotency genes are permitted and where network-inherent fluctuation or perturbations have no regulatory effect. We could verify that the induction of differentiation in a mESC culture, previously maintained under 2i conditions, can be consistently described through a two step differentiation sequence, in which the initial, potentially asynchronous downregulation of Nanog (and Rex1 as its read-out) is succeeded by final downregulations of Oct4 and Sox2, thus preventing a reversion into pluripotency.

## Supporting Information

File S1 (DOCX)
