# Supplementary material for: A Model-Based Analysis of Culture-Dependent Phenotypes of mESCs
Source: PLoS One. 2014 Mar 18;9(3):e92496. doi: 10.1371/journal.pone.0092496 (PMC3958526; doi:10.1371/journal.pone.0092496)
Supplement: File S1 — (DOCX) [file pone.0092496.s001.docx]

**Supplement**

**Parameter selection and adjustment**

We perform bifurcation and stability analysis on the deterministic system (i.e. without the TF-specific background noise) using the software tool xppaut (<http://www.math.pitt.edu/~bard/xpp/xpp.html>). From this analysis we derive a set of parameters, which can essentially change the existence and the level of stable steady states of a particular TF. However, these parameters do not act independently from each other, e.g. alterations in the transcription rates of a certain factor can be compensated by simultaneously changing its degradation rate. The same applies for the binding rates k. Due to the in-ability to precisely measure these rates experimentally, we fixed the degradation rates d_j_ (with j ∈ (OS,N,R,E)) and the binding rates k assuming the same intensities for all model components. Subsequently, we manually adjust the remaining parameters, i.e. the transcription rates s_i_ (with i ∈ (1,2,…,7)), the repression rate p and the TF-specific noise intensities σ_j_ (with j ∈ (OS,N,R) according to experimentally observed TF distributions (Chambers et al, 2007; Kalmar et al, 2009; Marks et al, 2012; Toyooka et al, 2008).

*LIF/serum scenario*

- The transcription rate s_4_, the repression rate p and the Nanog-specific noise intensity σ_N_ are adjusted such that single mESCs are capable to switch between two coexisting Nanog states (a NH and a NL state). In a large number of simulated mESCs a fraction of about 80% NH and 20% NL cells should be established (Chambers et al, 2007; Kalmar et al, 2009; Wray et al, 2010) (Figure 3B-D).
- The transcription rate s_3_ determines the Nanog concentration in the NL state. We adapt this rate in a way that the two expression peaks of the bimodal Nanog distribution (i.e. the maxima concentrations in both Nanog states) differ from each other by two orders of magnitude (Chambers et al, 2007; Kalmar et al, 2009; Wray et al, 2010) (Figure 3D).
- The transcription rates s_5_, s_6_ and the Rex1-specific noise intensity σ_R_ are adapted to fit the distribution of Rex1GFPd2 mESCs measured by flow cytometry (grey histogram in Figure 3E).
- Furthermore, the combined transcription rate s_1,2_ and the Oct4-Sox2-specific noise intensity $\sigma_{OS}$ are chosen such that concentrations of the heterodimer stay constantly high and establish a homogeneous (unimodal) distribution (Figure 3D).
- Moreover, we introduce a normalization factor n such that the original Nanog distribution is reestablished within 12-14 days starting from a pure NL subpopulation. This results in a normalized time scale τ = n*t with arbitrary unit.

*2i scenario*

All parameter values except the value of repression rate p have been taken over from the LIF/serum scenario.

- The repression rate p is set to zero (Figure 4B).

The default (culture condition independent) parameter set is given in Table S1.

*Differentiation scenario*

To model the differentiation process, the repression rate p is increased for each mESCs individually by a Gaussian process with positive (non-zero) mean, i.e. N(μ_p_,σ_p_). Thus, in the differentiation scenario rate p is time-dependent and slightly different for each cell. In order to fit the experimental qRT-PCR data (shown in Figure 6A) we

- reduce the turnover of Rex1 protein compared to self-renewing conditions, i.e. we multiply all regulatory rates of Rex1 (i.e. s_5_, s_6_ and d_R_) by a manually chosen factor f=0.02 and
- adjust the external differentiation signal Y and the inhibition rates i_k_ (with k ∈ (OS,N,R)) accordingly.

Applying these parameters, simulated Rex1 distributions at different time points after the initiation of differentiation as shown in Figure 6D are a direct result without any further adaption.

The parameter set used for the differentiation scenario is given in Table S2.

| Parameter | Value | Parameter | Value |
| --- | --- | --- | --- |
| s_1,2_ | 75* | d_O,S_ | 0.01^#^ |
| s_3_ | 0.1* | d_OS_ | 1^#^ |
| s_4_ | 40* | d_N_ | 1^#^ |
| s_5_ | 0.3* | d_R_ | 0.02^#^ |
| s_6_ | 2.8* | d_E_ | 1^#^ |
| s_7_ | 2* | σ_OS_ | 0.05 |
| k | 0.1 | σ_N_ | 0.115 |
| k_Y_ | 4 | σ_R_ | 0.05 |
| i_OS_ | 0.8 | σ_p_ | 0.05 |
| i_R_ | 12 | μ_p_ | 0.38 |
| i_N_ | 1.0 | Y | 1.0 |
|  |  |  |  |
| p | stochastic | n | 0.01 |

| Parameter | Value | Parameter | Value |
| --- | --- | --- | --- |
| s_1,2_ | 75* | d_O,S_ | 0.01^#^ |
| s_3_ | 0.1* | d_OS_ | 1^#^ |
| s_4_ | 40* | d_N_ | 1^#^ |
| s_5_ | 15* | d_R_ | 1^#^ |
| s_6_ | 140* | d_E_ | 1^#^ |
| s_7_ | 2* | σ_OS_ | 0.05 |
| k | 0.1 | σ_N_ | 0.115 |
| k_Y_ | 4 | σ_R_ | 0.05 |
| i_OS_ | 0 | i_R_ | 0 |
| i_N_ | 0 | Y | 0 |
|  |  |  |  |
| p | culture-dependent | n | 0.01 |

**Table S1 Default parameter set** (* molecules/time unit; ^#^1/time unit)

**Table S2 Differentiation parameter set** (* molecules/time unit; ^#^1/time unit)

**Initial conditions**

For both the LIF/serum and the 2i scenario we performed a long-run simulation using the respective parameter set in order to generate and subsequently store an individual pool of initial conditions for all four variables (i.e. [OS], [N], [R], [E]). If a cell population was simulated, the initial concentrations have been taken randomly from the previously simulated distributions.

For scenario-independent simulations studies like shown in Figure 2B and Figure 5A, high initial concentrations for each of the four variables (i.e. [OS] = 60, [N] = 40, [R] = 140, [E] = 1) have been used. According to the experimental setting, the differentiation scenario is initialized with the parameter set and initial conditions for the 2i scenario.

**Simulation procedure**

To compare flow cytometry measurements of TFs with the results of our model analysis, we simulate TF concentrations in a large number of individual cells per time point. Herein each cell represents an independent realization of the outlined, stochastic molecular model (e.g. Figure 3C and 4C). Simulated single cell trajectories for mESCs under LIF/serum conditions are illustrated in Figure S1.

After simulating a large number of cells (about 10.000 cells) with the parameter set given in Table 1, the concentrations [OS], [N] and [R] and their frequencies are determined at time point t=4320 (i.e. 3 days in virtual culture, e.g. Figure 3D and 4D). We shift the resulting Nanog distribution to match the experimentally observed high expression peaks under LIF/serum [[21](#_ENREF_21),[22](#_ENREF_22)]. The simulated distributions of Oct4-Sox2 and Rex1 are shifted to fit to the same scale.

In order to estimate residence times of single mESCs in the NH and the NL state and transition probabilities between both states under LIF/serum conditions, we simulate a large number of cells (5000 cells with the LIF/serum parameter set, i.e. p=15) for very long time periods (over 1051200 steps) and measure the total number of switches as well as the time cells spend in one of the two states. The resulting distributions of residence times for both states and the mean number of state transitions are shown in Figure S2 and S3.

To compare qRT-PCR measurements with our model results, we average simulated concentrations of Nanog and Rex1 at the same time points on which the experimentally measurements have been taken (Figure 6A).


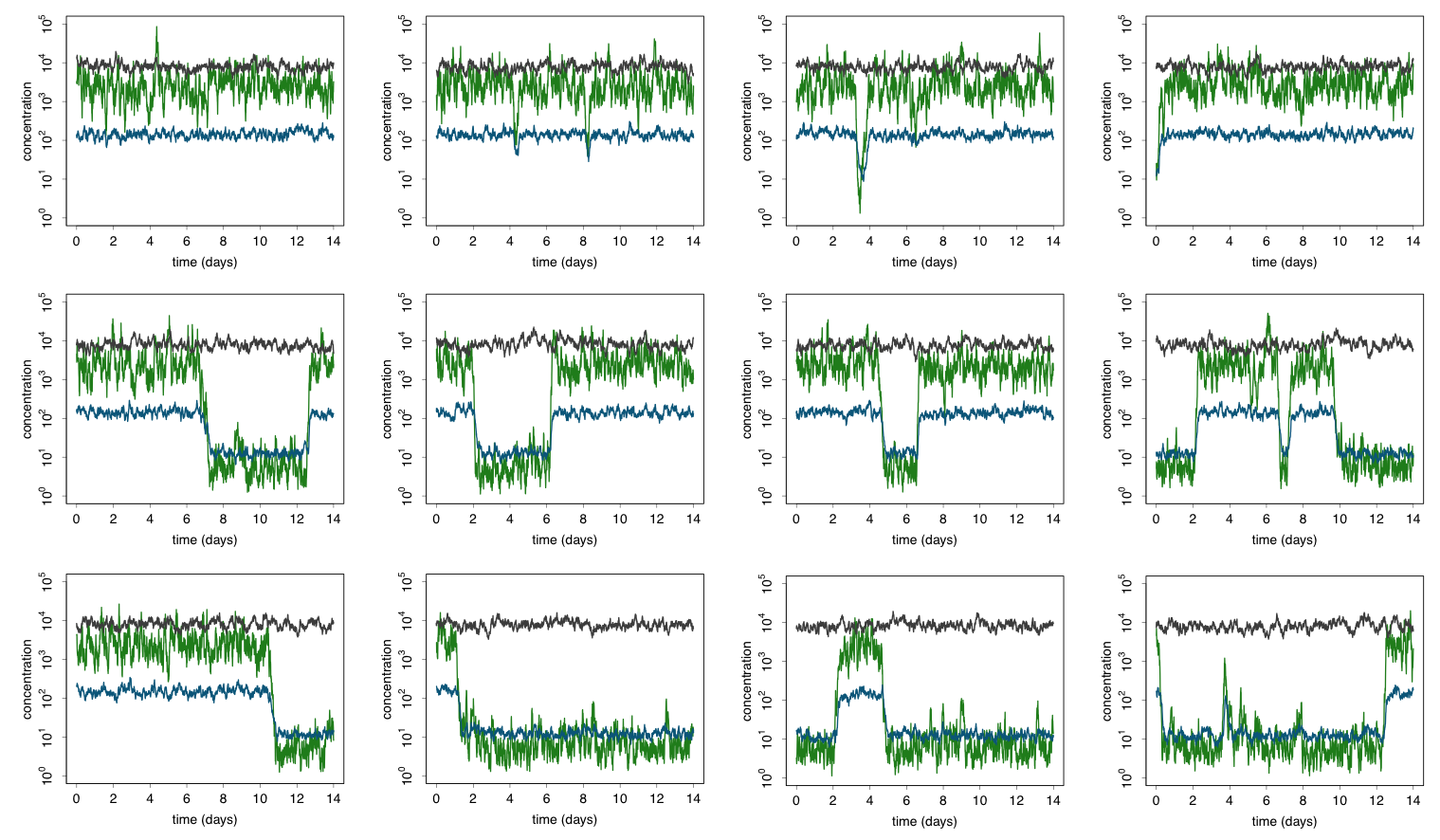


**Figure S1. Simulated TF concentrations of single mESCs in the LIF/serum scenario.** Panels show typical trajectories of Oct4-Sox2 (grey), Nanog (green) and Rex1 (blue) concentrations within single mESCs. The parameter set is given in Table S1 with p=15.


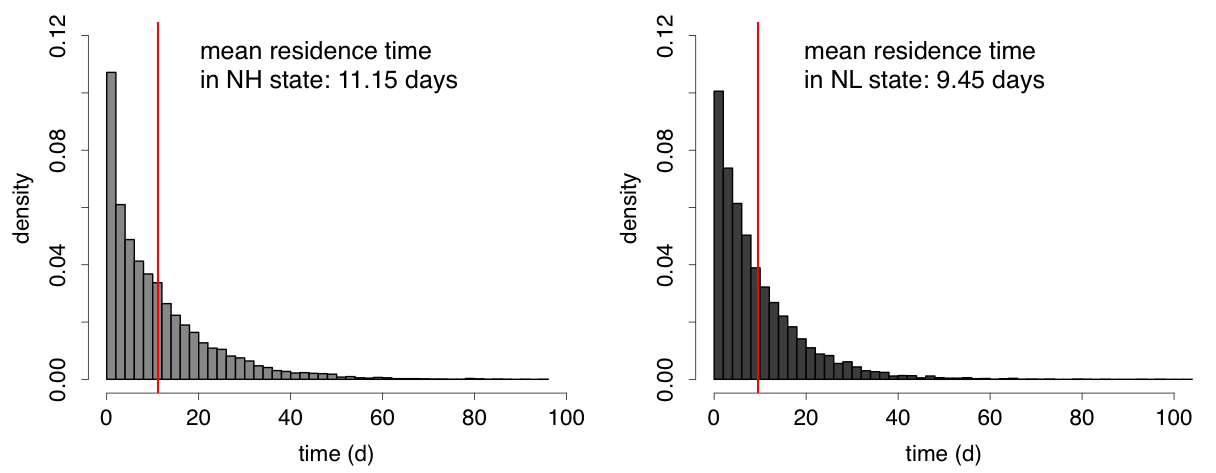


**Figure S2. Simulated residence times in the NH and the NL state.** In the LIF/serum scenario the residence times of mESCs in the NH and the NL state follow an exponential distribution. The mean residence time in the NH state is about 11 days (maximum: 98.06 days, median: 7.65 days). The mean residence time in the NL state is about 9 days (maximum: 104.5 days, median: 6.54).


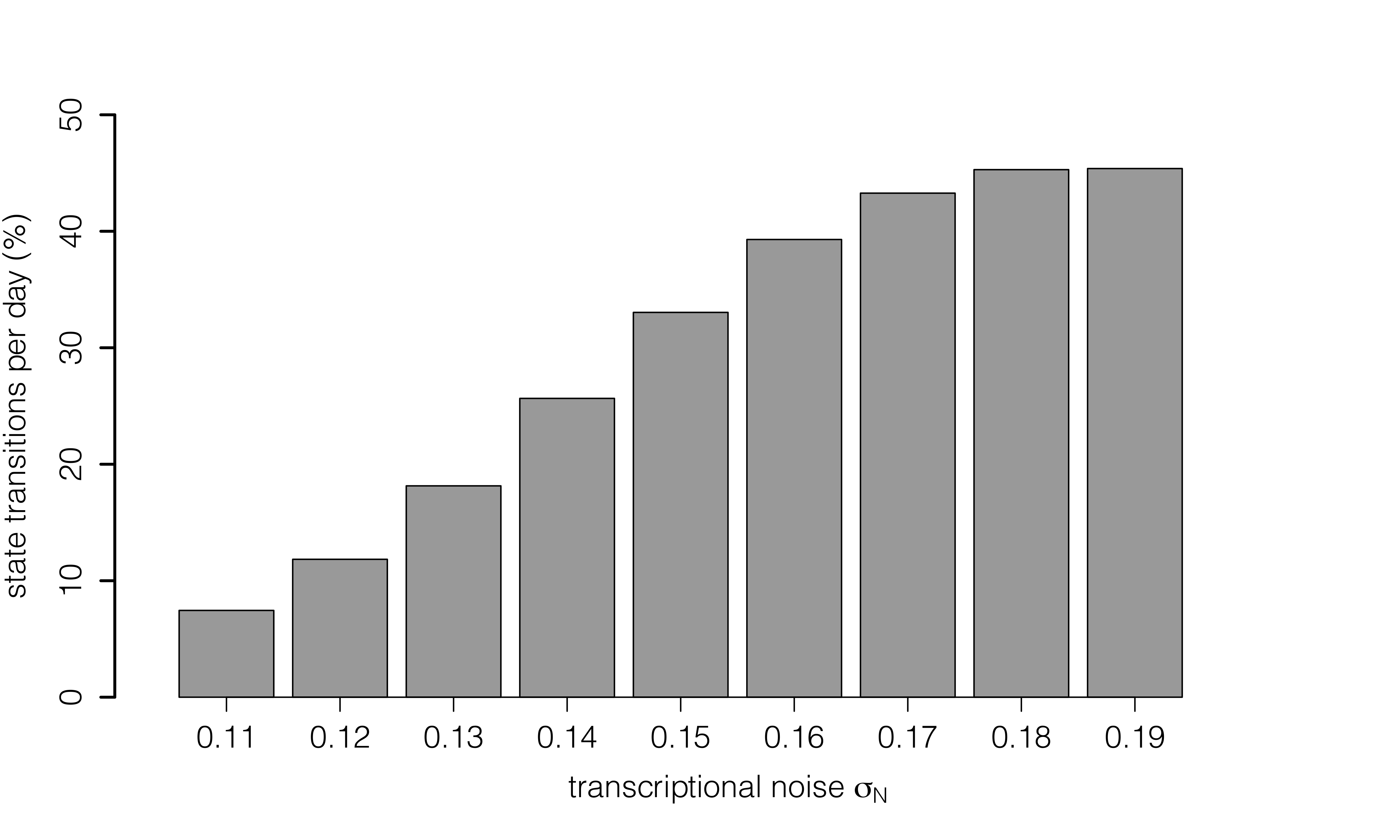
**Figure S3. Dependency of the number of state transitions on the noise intensity** σ_N._ The mean number of state transitions per day is shown for different values of σ_N_**.**

**Sensitivity analysis**

The Nanog expression is primarily determined by its own autoregulation (rate s_4_), by the activating input from Oct4-Sox2 (rate s_3_), by the inhibition of FGF4/Erk signalling (rate p) and by the transcriptional background noise σ_N_. Here we study how these rates “cooperate” and affect the fraction of NL cells (given in %) in a mESC population maintained under LIF/serum conditions (i.e. with the parameter given in Table S1 and p=15).


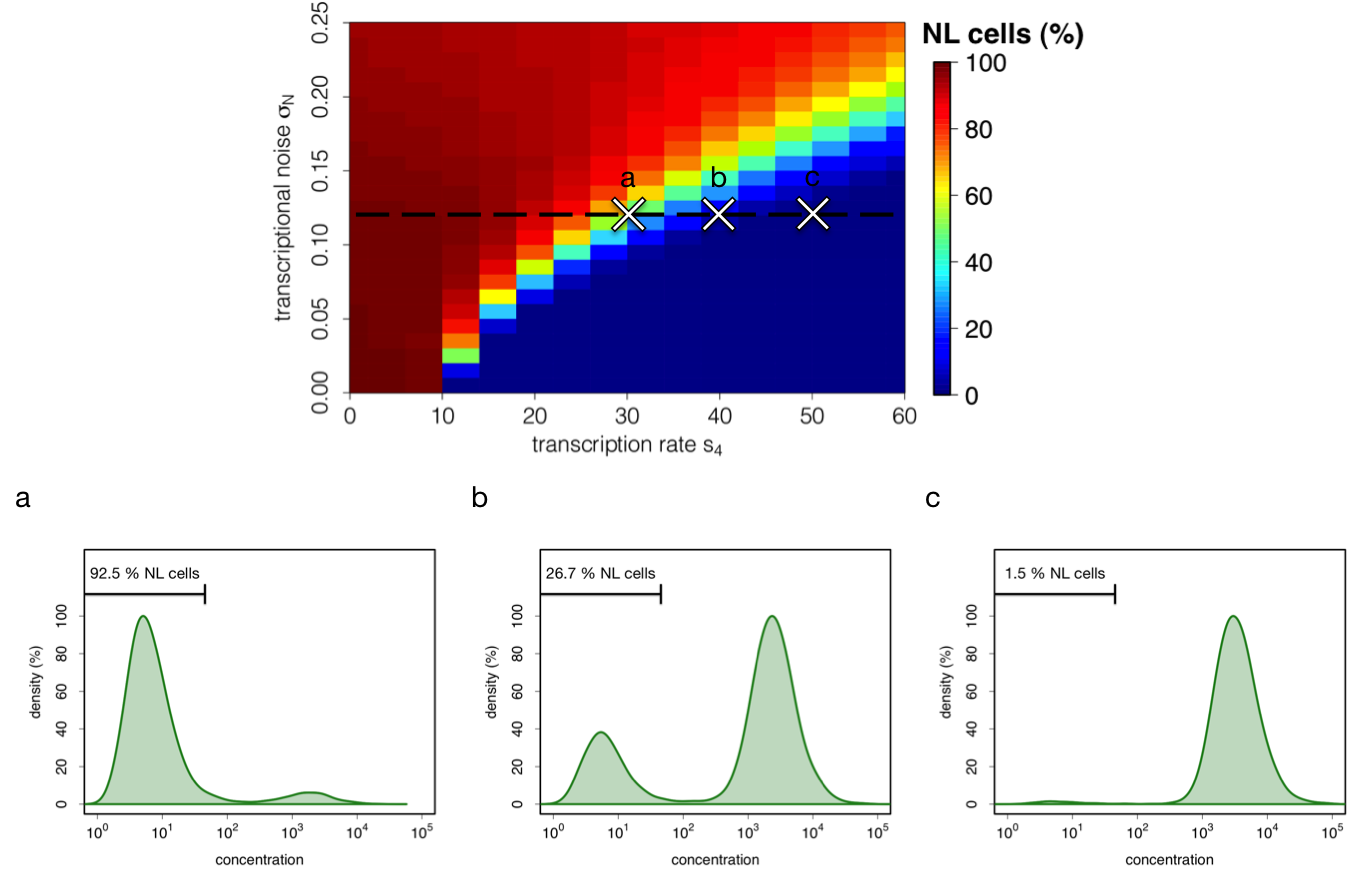
The dependency of the fraction of NL cells on the strength of Nanog autoactivation s_4_ and the transcriptional background noise σ_N_ is illustrated in Figure S4. Given a constant noise intensity (σ_N_ = 0.12, parameter choice indicated by the dotted black line in the heat map), an increase in the transcription rate s_4_ reduces the proportion of NL cells. Example distributions for selected values of s_4_ (parameter choice indicated by white crosses in the heat map) are illustrates below (Figure S4 a-c).

**Figure S4. Relationship between the fraction of NL cells and model parameters s_4_ and σ_N_.** For any constant background noise (e.g. vertical, black line at σ_N_=0.12) an increase of s_4_  enhances the fraction of NH cells (a-c).

The Oct4-Sox2-mediate Nanog activation by s_3_ determines the Nanog concentration in the NL state. Increasing this rate shifts the NL state towards the NH state and thus decreases the fraction of NL cells as shown in Figure S5.

**
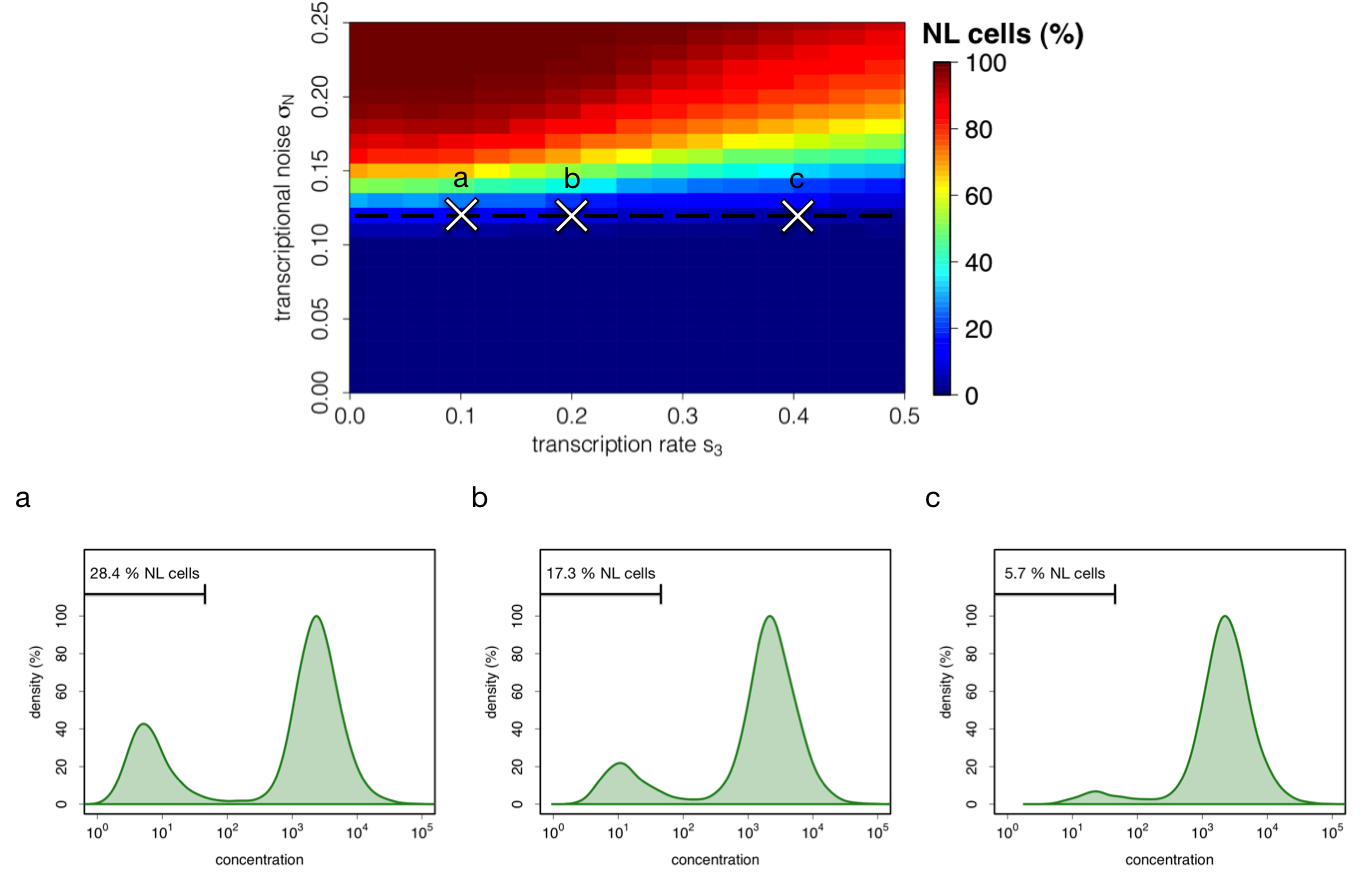
Figure S5. Relationship between the fraction of NL cells and model parameters s_3_ and.** Given a certain, constant degree of noise (e.g. vertical, black line at σ_N_=0.12) and a constant transcription rate s_4_ (e.g. s_4_=40), an increase in transcription rate s_3_ rate shifts the NL state towards the NH state and thus the fraction of NL cells decreases (a-c).

**Heterogeneity during mESC differentiation**

In order to quantify the predicted heterogeneity during the differentiation process, we calculated and plotted the median concentrations and the interquartile ranges (IQR) of Nanog and Rex1 at different time points after the induction of differentiation (Figure S6). The variability of Nanog is predicted to be highest around 14h after the inducing differentiation (IQR of 10^2.4^ with 50% of the cells showing a Nanog concentration between 10^0.6^ and 10^3^), while the variability of Rex1 is highest around 26h (IQR of 10^1^ with 50% of the cells showing a Rex1 concentration between 10^0.1^ and 10^2.1^).


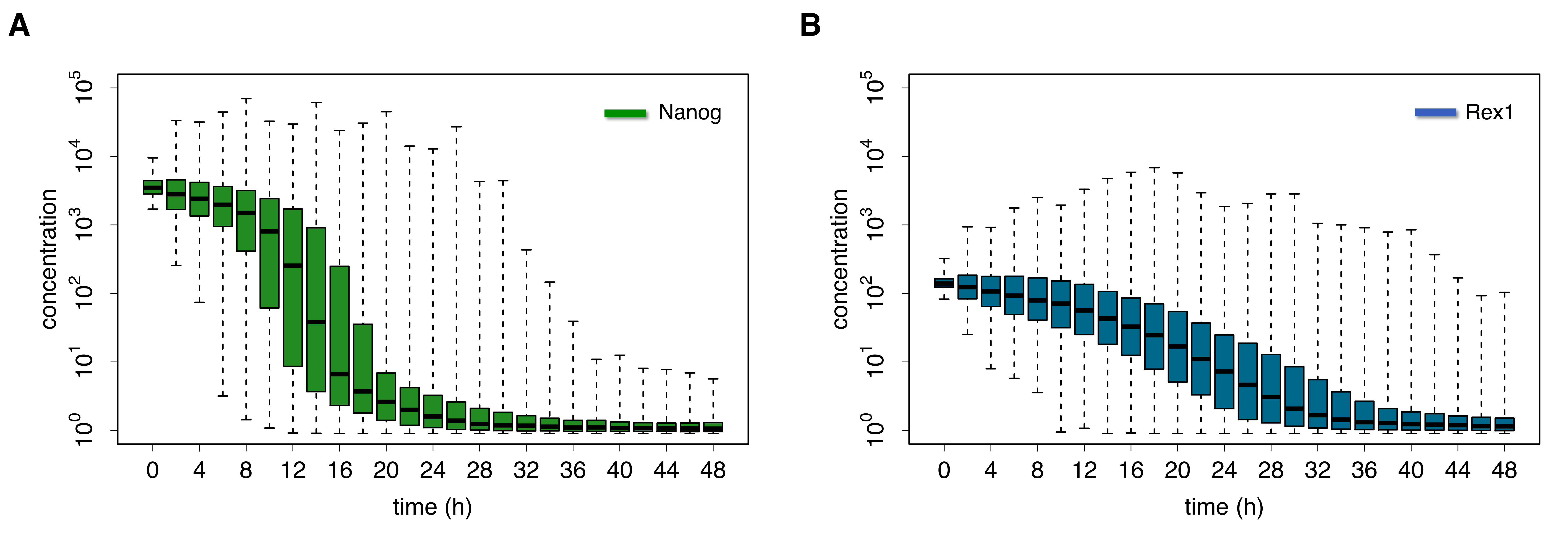
**Figure S6. Simulated TF concentrations during differentiation.** Boxplots show Nanog (A) and Rex1 (B) expression levels in a cell population at different time points during differentiation. The thick line in the middle of a box indicates the median concentration at this time point, while the length of a box illustrates the interquartile range (IQR) as a measure for variability. The whiskers depict the 1.5 x IQR
